# The association between serum selenium levels and pathological features of papillary thyroid cancer in 284 patients

**DOI:** 10.3389/fendo.2023.1242250

**Published:** 2023-11-03

**Authors:** Shenghui Ge, Junyu Zhao, Jinming Yao, Hang Fu, Yutian Tian, Yuanyuan Shan, Mengli Sun, Jing Feng, Jianjun Dong, Lin Liao

**Affiliations:** ^1^ Department of Endocrinology and Metabology, The First Affiliated Hospital of Shandong First Medical University & Shandong Provincial Qianfoshan Hospital, Ji-nan, China; ^2^ College of Traditional Chinese Medicine, Shandong University of Traditional Chinese Medicine, Ji-nan, China; ^3^ Division of Endocrinology, Department of Internal Medicine, Qilu Hospital of Shandong University, Ji-nan, China; ^4^ Department of Endocrinology and Metabology, The First Affiliated Hospital of Shandong First Medical University & Shandong Provincial Qianfoshan Hospital, Shandong Key Laboratory of Rheumatic Disease and Translational Medicine, Shandong Institute of Nephrology, Ji-nan, China

**Keywords:** papillary thyroid cancer, pathological features, nutrition, selenium, serum

## Abstract

**Objective:**

The relationship between serum selenium levels and papillary thyroid cancer (PTC), especially the pathological features, still remains controversial. We conducted this study to investigate the relationship between serum selenium levels and PTC in a Chinese population.

**Methods:**

Cross-sectional data of 284 patients with PTC were collected from the First Affiliated Hospital of Shandong First Medical University. The general clinical characteristics, serum selenium levels, and tumor pathological features were described in PTC. The association between serum selenium levels and pathological features in PTC was analyzed using SPSS 26.0 statistical software.

**Results:**

Our results showed that the median serum selenium level was 79.15 μg/L (IQR: 71.00 - 86.98 μg/L) in PTC patients. Serum selenium levels were lower in females than males (*p* = 0.035). Serum selenium levels were negatively correlated with the number of lymph node metastases (*p* = 0.048). High serum selenium (*OR* = 0.397, 95%*CI*: 0.217 - 0.725) and diastolic blood pressure (*OR* = 1.028, 95%*CI*: 1.005 - 1.051) were related factors for the incidence of bilateral tumors. High serum selenium (*OR* = 0.320, 95%*CI*: 0.166 - 0.617) and diastolic blood pressure (*OR* = 1.066, 95%*CI*: 1.031 - 1.103) were related factors for tumor multifocal incidence.

**Conclusions:**

The serum selenium levels of PTC patients in females were lower than males. High serum selenium levels might be a protective factor in PTC patients. Further research is necessary to better understand the influence of selenium on PTC progression.

## Introduction

Nowadays cancer is the second leading cause of death, severely affecting people’s quality of life and longevity (1). Thyroid cancer is the most prevalent malignant tumor of the endocrine system, and its incidence has been on the rise in recent years (2, 3). Notably, papillary thyroid cancer (PTC) accounts for a vast majority of cases (4). Despite a favorable prognosis for most patients following systematic treatment, a subset experiences relapse and unfavorable outcomes (5). Previous research has identified large tumor diameters, extrathyroidal extension, and distant lymph node metastases as significant predictors of thyroid cancer recurrence and mortality (6, 7). While some risk factors for thyroid cancer have been predicted, most are uncontrollable, including advanced age, gender, race and family history. Therefore, the exploration of controllable risk factors becomes crucial for the prevention and treatment of thyroid cancer.

Selenium, an essential trace element, plays a pivotal role in maintaining homeostasis. Selenium manifests its anti-inflammatory, antioxidant, antiviral, and anticancer characteristics through its involvement in the synthesis of diverse selenoproteins (8–11). At present, a minimum of 25 selenoproteins with diverse biological functions have been identified (12). Maintaining proper selenium status is crucial for human health, as deficiency is associated with increased risks of infertility, autoimmune imbalance, inflammation, and cancer. Alternatively, excessive serum selenium levels may lead to hyperlipidemia, hyperinsulinemia, type 2 diabetes mellitus, and atherosclerosis (13).

Recently, the relationship between selenium and thyroid cancer has garnered attention. The thyroid has a significantly higher concentration of selenium than other organs (14). As oxidative stress may lead to cellular mutation and gene variation, selenoproteins have been considered to prevent the development of cancer through their antioxidant effects (15). However, studies on selenium and tumor risk have yielded inconsistent results. Most studies have found that low selenium appears to be associated with the incidence of thyroid cancer (16–19). In the study by Baltaci et al., patients with thyroid cancer demonstrated lower serum selenium levels compared to those without thyroid cancer (16). Kucharzewski et al. found that blood selenium concentrations in patients with thyroid cancer were lower than those in controls (19). In a case-control trial, serum selenium levels were inversely associated with the risk of thyroid cancer (17). Additionally, a meta-analysis involving 1291 subjects showed that blood selenium levels in thyroid cancer were lower than those in controls (18). However, a Polish study found no significant differences in serum selenium levels between patients with thyroid cancer and non-thyroid cancer patients (20). Moreover, no association between selenium intake and thyroid cancer was observed in a large prospective cohort study in the United States (14). In the study by Jonklaas et al., it was noted that serum selenium levels in patients with thyroid cancer were not significantly reduced, but they were negatively correlated with the disease stage (21).

Currently, studies focus on the relationship between serum selenium levels and the incidence of thyroid cancer, yet they lack further analysis of pathological features. Furthermore, most studies included other pathological types of thyroid cancer and did not study PTC separately. Therefore, we investigated serum selenium levels and pathological features in PTC patients to study the relationship between serum selenium levels and pathological features.

## Materials and methods

### Trial design and participants

This cross-sectional study was extracted from a larger prospective study. Patients with PTC who were treated at the First Affiliated Hospital of Shandong First Medical University (Qianfoshan Hospital of Shandong Province) from January 1, 2021 to December 31, 2022 were included in this study. All patients have completed the surgical pathological details, and serum selenium levels were detected during hospitalization.

### Inclusion criteria

(1) Research period: January 1, 2021 to December 31, 2022.
(2) Surgical method: total thyroidectomy with or without lymph node dissection.
(3) Histopathological results: PTC was clearly diagnosed, and complete pathological details were recorded.
(4) Complete the detection of serum selenium index.

### Exclusion criteria

(1) It is known that other primary tumors have metastasized to the thyroid gland.
(2) Those who have taken selenium preparations or selenium-enriched drugs in the past year.
(3) Patients who are pregnant or breastfeeding.
(4) Acute complications of diabetes (diabetic ketoacidosis, hyperglycemia and hyperosmolar state).
(5) Significant liver function abnormalities, defined as aspartate aminotransferase > 2 × ULN (upper limit of normal range) and/or alanine aminotransferase > 2 × ULN, and/or total bilirubin > 34umol/L or previous infectious hepatitis seropositive evidence.
(6) Patients with renal insufficiency (eGFR < 60ml/min/1.73m²).
(7) Patients with severe autoimmune diseases (rheumatoid, systemic lupus erythematosus and other diseases).

### Data collection

(1) General clinical data, including name, age, gender, height, weight, waist, systolic blood pressure (SBP), diastolic blood pressure (DBP) and thyroid related indicators.
(2) Serum selenium levels: Inductively coupled plasma mass spectrometry (ICP-MS), collected by the Laboratory Department of the First Affiliated Hospital of Shandong First Medical University (Qianfoshan Hospital in Shandong Province), tested and provided by Guangzhou Jinyu Medical Laboratory Institute Report.
(3) Details of thyroid surgery (total thyroidectomy, with or without lymph node dissection), histopathological results (pathological type, tumor size, number of metastatic lymph nodes, whether the lesion is unilateral or bilateral, whether the lesion is multifocal, or whether the lesion is invasive) capsule, and presence or absence of metastasis), and TNM (tumor, node, metastasis) staging based on pathological details (American Joint Committee on Cancer, AJCC 8th ed.).

### Statistical methods

All data were statistically analyzed using SPSS 26.0 statistical software. Describe patient demographics, clinical characteristics, and blood parameters using simple summary statistics. The Kolmogorov-Smirnov test was performed on the enumeration data, and the data that conformed to the normal distribution were expressed as mean ± standard deviation, and the data that did not conform to the normal distribution were expressed as the median (interquartile range). Univariate analysis was performed by t test or Wilcoxon signed rank test, and Logistic regression analysis was used for further analysis of variables with statistical differences. The independent samples Kruskal-Wallis rank sum test was used for comparison among multiple groups. Spearman or Pearson correlation analysis was used for bivariate analysis. All tests were two-sided, and *p* < 0.05 indicated a statistically significant difference.

## Results

### Patient characteristics

A total of 284 PTC patients were included in the study. The demographic and clinical data were shown in **Table 1**. Among the 284 patients, 85 were males (85/284, 29.93%) and 199 were females (199/284, 70.07%). The median age was 44 years (IQR: 35.00 - 52.00 years). The median BMI was 25.71 kg/m^2^ (23.14 - 28.46 kg/m^2^). According to the standards established by the China Obesity Working Group Data Summary and Analysis Collaborative Group, 1 patient (1/284, 0.35%) was underweight (<18.5 kg/m^2^), 91 patients (91/284, 32.04%) were normal weight, and 113 patients (113/284, 39.79%) were overweight (24-27.9 kg/m^2^), and 79 patients (79/284, 27.82%) were obese (≥28 kg/m^2^). The median height was 163.00 cm (IQR: 160.00 - 170.00 cm), and the median weight was 69.5 kg (IQR: 60.25 - 80.00 kg). Waist was recorded in 282 patients, with an average of 90.90 cm. The median SBP and DBP in patients were 123.00 mmHg (IQR: 114.00 - 135.00 mmHg) and 80.48 mmHg (IQR: 73.00 - 88.75 mmHg), respectively. The clinical data of PTC patients with different genders were shown in **Table S1**. Females exhibited higher levels of thyroid antibodies compared to males, while males demonstrated a higher BMI and blood pressure than females.

**Table 1:** Demographic and clinical data observed in the cross-section study.

Subjects	Data
Onset (Year)	44.00 (35.00 - 52.00)
Gender (N, %)	
Male	85 (29.93%)
Female	199 (70.07%)
Waist (cm)	90.90 ± 10.90
BMI (kg/m^2^)	25.71 (23.14 - 28.46)
Underweight (N, %)	1 (0.35%)
Normal weight (N, %)	91 (32.04%)
Overweight (N, %)	113 (39.79%)
Obese (N, %)	79 (27.82%)
SBP (mmHg)	123.00 (114.00 - 135.00)
DBP (mmHg)	80.48 (73.00 - 88.75)
FT3 (pmol/L)	4.94 (4.62 - 5.41)
FT4 (pmol/L)	17.73 (16.50 - 19.84)
TSH (uIU/mL)	1.85 (1.16 - 2.64)
TPOAB (IU/mL)	13.08 (10.04 - 20.13)
TGAB (IU/mL)	20.24 (15.72 - 49.35)

BMI, body mass index; SBP, systolic blood pressure; DBP, diastolic blood pressure; FT3, free triiodothyronine; FT4, free thyroxine; TSH, thyroid stimulating hormone; TPOAB, thyroidperoxidase antibodies; TGAB, thyroglobulin antibody.

### Characteristics of serum selenium levels in PTC patients

**Figure 1:**
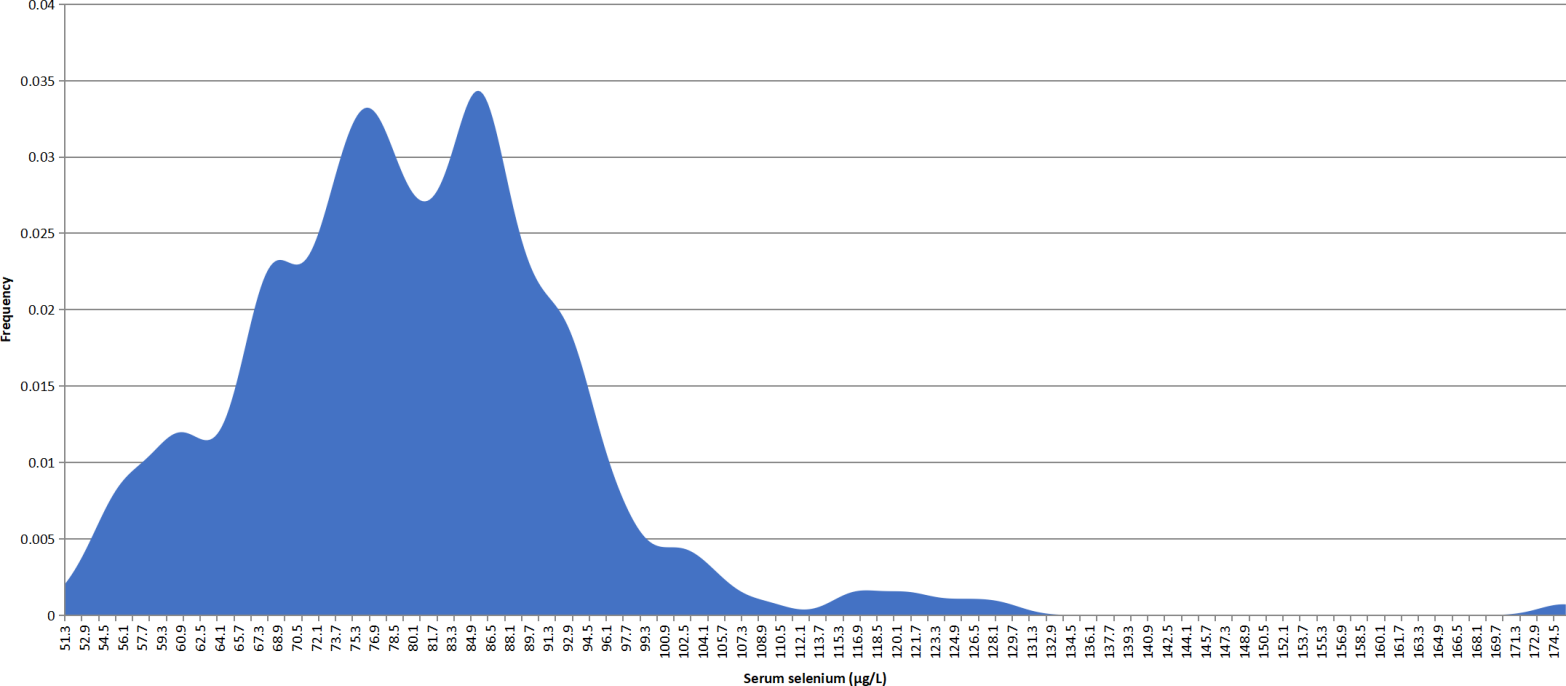
Overall characteristics of serum selenium levels in PTC patients.

**Figure 2:**
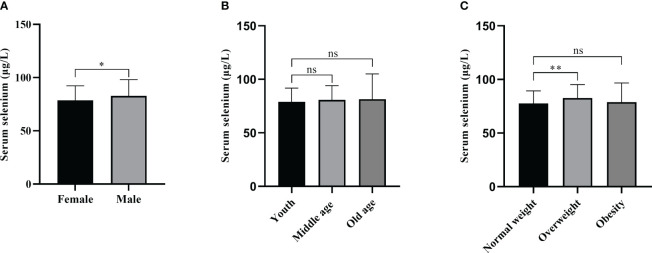
Serum selenium levels in PTC patients with different sexes, ages, and BMIs. **(A)** Serum selenium levels in PTC patients with different genders. **(B)** Serum selenium levels of PTC patients with different ages. **(C)** Serum selenium levels in PTC patients with different BMIs. ns, not significant; *, *p* < 0.05; **, *p* < 0.01.

The distribution characteristics of serum selenium levels were shown in **Figure 1**. The median serum selenium level was 79.15 μg/L (IQR: 71.00 - 86.98 μg/L). The serum selenium level of 232 patients (232/284, 81.69%) was below 90 μg/L. There were 199 female patients and 85 male patients. The serum selenium level was 78.30 μg/L (IQR: 68.80 - 86.30 μg/L) in female patients and 82.20 μg/L (IQR: 74.80 - 88.65 μg/L) in male patients. As shown in **Figure 2A**, the serum selenium level in female patients was lower than that in male patients (*p* = 0.035). Age categories for patients were classified according to the WHO criteria: 144 patients in the young group (<45 years old), 116 cases in the middle-aged group (45-59 years old), and 24 cases in the old group (≥60 years old). The serum selenium level was 79.05 μg/L (IQR: 69.65 - 86.60 μg/L) in the young group, 79.35 μg/L (IQR: 72.85 - 88.43 μg/L) in the middle-aged group, and 77.70 μg/L (IQR: 70.83 - 86.43 μg/L) in the elderly group. There was no significant difference among three groups in serum selenium levels (*p* = 0.944) (**Figure 2B**). BMI categories for patients were classified according to the China Obesity Working Group Data Summary and Analysis Collaborative Group: 91 patients were normal weight (18.5-23.9 kg/m^2^), 1 patient was underweight (<18.5 kg/m^2^), 113 patients were overweight (25-27.9 kg/m^2^), and 79 patients were obesity (≥28 kg/m^2^). Since only one patient was underweight, it was not included in the comparison. The serum selenium level in normal weight patients was 78.30 μg/L (IQR: 69.60 - 85.20 μg/L), the serum selenium level in overweight patients was 82.60 μg/L (IQR: 75.15 - 91.15 μg/L) and obesity patients was 76.10 μg/L (IQR: 68.50 - 86.20 μg/L). Serum selenium levels in overweight patients were significantly higher than those in normal weight groups (*p* < 0.01) (**Figure 2C**). The correlation between serum selenium levels and thyroid-related indicators was presented in **Table S2**. The results showed that there was no significant correlation between selenium and thyroid related indicators.

### Pathological features in PTC patients

The detailed pathological characteristics of PTC patients were shown in **Table 2**. There were 142 patients (142/284, 50.00%) with unilateral tumors and 137 patients with bilateral lesions (137/284, 48.24%). There were 168 patients (168/284, 59.15%) with multifocal tumors, and 102 patients (102/284, 35.92%) with single tumor tumors. There were 159 (159/284, 55.99%) patients with thyroid capsule invasion. The median tumor size was 1.10 cm (IQR: 0.70 - 1.60 cm). The median number of positive lymph node metastases was 4 (IQR: 1.00 - 8.00). The results of T staging showed that most of the patients were in stage I, including 127 patients in stage Ia (127/284, 44.72%) and 79 patients in stage Ib (79/284, 27.82%). N staging results showed that more than half of the patients were stage Ia.

**Table 2:** Pathological features of enrolled patients.

Subjects	Data
Bilateral	
Unilateral lesion	142 (50.00%)
Bilateral lesion	137 (48.24%)
Not mentioned	5 (1.76%)
Multifocality	
Yes	168 (59.15%)
No	102 (35.92%)
Not mentioned	14 (4.93%)
Capsule invasion	
Yes	159 (55.99%)
No	30 (10.56%)
Not mentioned	95 (33.45%)
Tumor size (cm)	1.10 (0.70 - 1.60)
Number of metastatic lymph nodes	4.00 (1.00 - 8.00)
TNM staging (AJCC 8th edition)	
T	
Ia	127 (44.72%)
Ib	79 (27.82%)
II	28 (9.86%)
IIIa	3 (1.06%)
IIIb	11 (3.87%)
Iva	9 (3.17%)
IVb	4 (1.41%)
X	23 (8.10%)
N	
0	51 (17.96%)
Ia	159 (55.99%)
Ib	71 (25.00%)
NA	3 (1.06%)

NA, not available.

### Serum selenium levels and pathological characteristics in PTC patients

The association between serum selenium levels and pathological features in PTC patients was analyzed by Spearman correlation analysis (**Table 3**). Serum selenium levels were negatively correlated with the number of lymph node metastasis (*r* = -0.121, *p* = 0.048). There was no significant correlation with tumor T stage (*p* = 0.436), N stage (*p* = 0.811) and tumor maximum diameter (*p* = 0.795).

**Table 3:** Spearman correlation analysis of serum selenium levels and pathological features in PTC patients.

Subjects	Serum selenium	
	r	*p*
TNM staging-T	-0.048	0.436
TNM staging-N	-0.015	0.811
Tumor size (cm)	0.016	0.795
Metastatic lymph nodes	-0.121	0.048*

**Figure 3:**
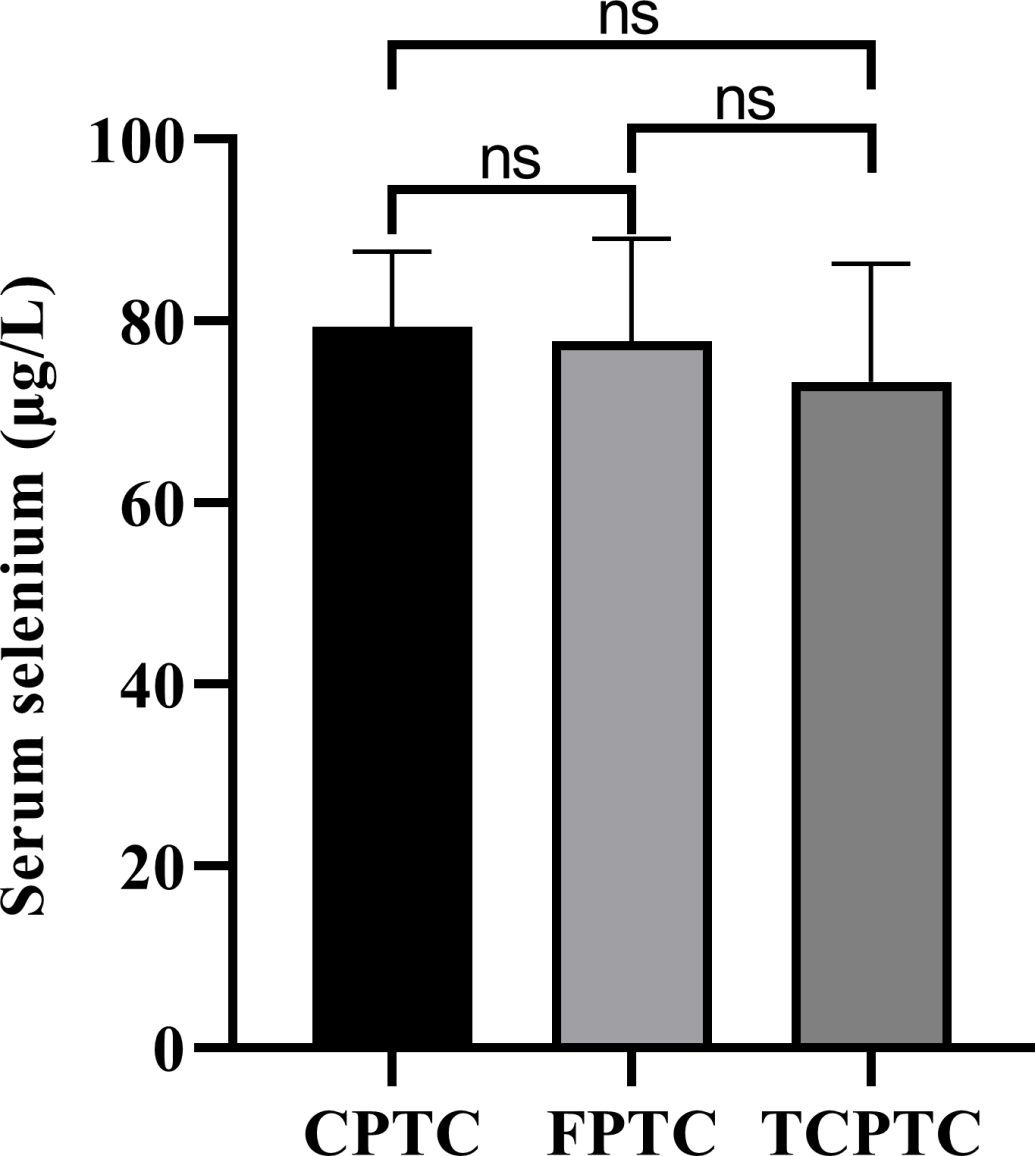
Serum selenium levels of different pathological subtypes in PTC patients. CPTC, common papillary thyroid cancer; FPTC, follicular papillary thyroid cancer; TCPTC, tall cell papillary thyroid cancer. ns, not significant.

The serum selenium levels of different pathological subtypes in PTC patients were shown in **Figure 3**. There were 270 patients with classical subtype PTC, 7 patients with follicular subtype PTC, and 7 patients with hypercellular subtype PTC. There was no significant difference in serum selenium levels among patients with different pathological subtypes of PTC (*p* = 0.860).

**Figure 4:**
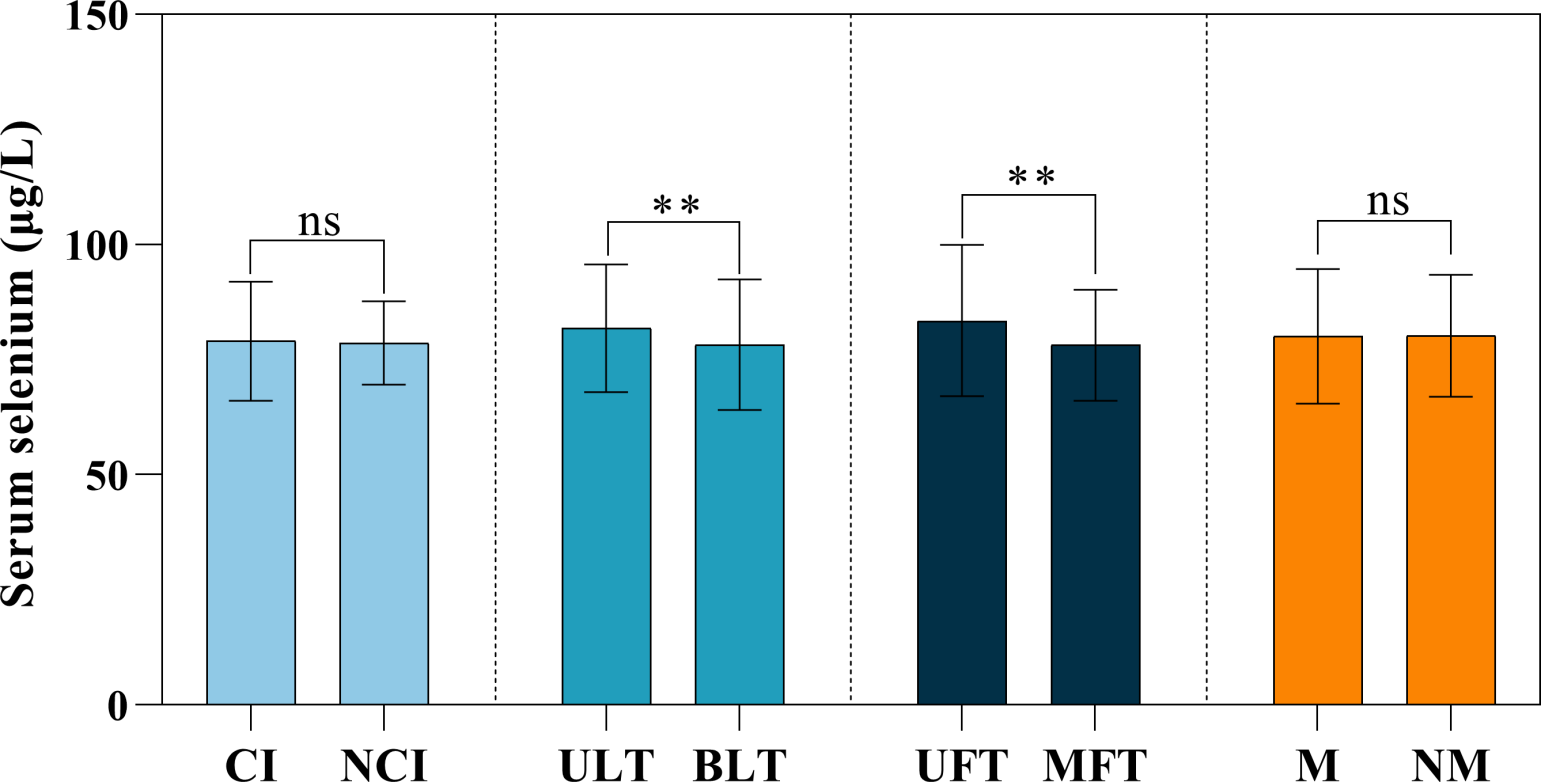
Serum selenium levels among different pathological features in PTC patients. CI, capsule invasion; NCI, no capsule invasion; ULT, unilateral tumor; BLT, bilateral tumor; UFT, unifocal tumor; MFT, multifocal tumor; M, metastasis; NM, no metastasis. ns, not significant; **, *p* < 0.01.

The comparison of serum selenium levels in PTC patients with different pathological features was shown in **Figure 4**. Serum selenium levels in patients with bilateral tumors were significantly lower than those in patients with unilateral tumors (*Z* = -2.871, *p* = 0.004). Serum selenium levels in patients with multifocal tumors were significantly lower than those in patients with unifocal tumors (*Z* = -4.120, *p* < 0.001). There was no statistical difference in serum selenium levels of patients regardless of membrane invasion (*t* = -0.172, *p* = 0.864) and lymph node metastasis (*t* = 0.472, *p* = 0.637). The comparison of serum selenium levels in common papillary thyroid cancer patients with different pathological features was shown in **Figure S1**.

### Factors related to unilateral and bilateral tumors in PTC patients

The status of unilateral or bilateral tumors in PTC patients was presented in **Table 4**. Univariate analysis showed that there were statistical differences in serum selenium level (*p* = 0.004), BMI (*p* = 0.033) and DBP (*p* = 0.018), while age (*p* = 0.647), height (*p* = 0.971), Waist (*p* = 0.051) and SBP (*p* = 0.188) were not statistically different.

**Table 4:** Comparison of data between unilateral tumor group and bilateral tumor group in PTC patients.

Subject	Unilateral tumor	Bilateral tumors	t/Z	*p*
Number (%)	142 (50.90)	137 (49.10)	-	-
Age (years)	45.00 (35.00 - 52.00)	43.00 (35.00 - 52.50)	-0.458	0.647
SSL (μg/L)	82.70 (72.55 - 89.43)	76.90 (70.05 - 85.05)	-2.871	0.004
BMI (kg/m^2^)	25.52 ± 3.91	26.66 ± 3.90	-2.436	0.015
Height (cm)	163.00 (160.00 - 170.00)	164.00 (160.00 - 170.00)	-0.036	0.971
Weight (kg)	66.50 (59.00 - 78.50)	70.00 (62.00 - 81.00)	-2.129	0.033
Waist (cm)	88.00 (83.00 - 97.00)	92.00 (84.25 - 98.75)	-1.949	0.051
SBP (mmHg)	122.00 (112.75 - 132.25)	124.00 (114.50 - 138.00)	-1.316	0.188
DBP (mmHg)	78.00 (71.75 - 86.00)	80.00 (74.00 - 92.00)	-2.364	0.018
FT3 (pmol/L)	4.92 (4.56 - 5.19)	5.07 (4.69 - 5.87)	-0.107	0.915
FT4 (pmol/L)	17.59 (15.40 - 20.12)	18.06 (16.97 - 19.65)	-0.721	0.471
TSH (uIU/mL)	2.04 (1.14 - 3.00)	1.49 (1.26 - 2.42)	-1.477	0.140
TPOAB (IU/mL)	13.08 (10.01 - 20.00)	12.67 (10.07 - 20.49)	-0.398	0.690
TGAB (IU/mL)	19.79 (15.06 - 44.08)	21.30 (16.03 - 55.02)	-0.820	0.412

SSL, serum selenium level; BMI, body mass index; SBP, systolic blood pressure; DBP, diastolic blood pressure; FT3, free triiodothyronine; FT4, free thyroxine; TSH, thyroid stimulating hormone; TPOAB, thyroidperoxidase antibodies; TGAB, thyroglobulin antibody.

Statistically significant variables in univariate analysis (serum selenium level, BMI, DBP) were included in multivariate analysis (**Table 5**). High serum selenium (*OR* = 0.387, 95%*CI*: 0.212 - 0.706) and DBP (*OR* = 1.029, 95%*CI*: 1.006 - 1.053) were the relevant factors for the incidence of bilateral tumors.

**Table 5:** Multivariate logistic retrospective analysis of factors related to unilateral and bilateral tumor incidence in PTC patients.

Subject	B	S.E.	Wald	*p*	*OR*	95%*CI*
SSL (μg/L)						
Low	-	-	-	-	1	-
Middle	-0.205	0.304	0.455	0.500	0.815	0.449 - 1.478
High	-0.924	0.307	9.027	0.003	0.397	0.217 - 0.725
BMI (kg/m^2^)	0.048	0.034	2.042	0.153	1.049	0.982 - 1.121
DBP (mmHg)	0.028	0.012	5.690	0.017	1.028	1.005 - 1.051

Serum selenium levels were categorized into three grades: Low: 51.3 - 74.4 μg/L (n = 93), Middle: 74.5 - 85.3 μg/L (n = 93), and High : 85.4 - 175.3 μg/L (n = 93); BMI, body mass index; DBP, diastolic blood pressure. SSL, serum selenium level.

### Factors related to single and multifocal tumors in PTC patients

The status of single or multifocal tumors in PTC patients was presented in **Table 6**. Univariate analysis showed that there were statistical differences in serum selenium level (*p* = 0.004), BMI (*p* = 0.033) and DBP (*p* = 0.018), while age (*p* = 0.647), height (*p* = 0.971), Waist circumference (*p* = 0.051) and SBP (*p* = 0.188) were not statistically different.

**Table 6:** Comparison of data between single-focal tumor group and multi-focal tumor group in PTC patients.

Subject	Unilateral tumor	Bilateral tumors	t/Z	*p*
Number (%)	102 (37.78)	168 (62.22)	-	-
Age (years)	43.00 (33.75 - 52.00)	45.00 (36.00 - 53.00)	-1.120	0.263
SSL (μg/L)	85.05 (74.88 - 92.15)	76.90 (69.43 - 85.25)	-4.120	<0.001
BMI (kg/m^2^)	25.43 ± 4.10	26.44 ± 3.89	-2.017	0.045
Height (cm)	163.00 (159.75 - 170.00)	164.00 (160.00 - 170.00)	-0.256	0.798
Weight (kg)	66.00 (57.38 - 79.63)	70.00 (62.00 - 80.00)	-1.940	0.052
Waist (cm)	90.19 ± 11.41	91.43 ± 10.58	-0.906	0.366
SBP (mmHg)	122.16 ± 15.85	126.64 ± 17.43	-2.120	0.035
DBP (mmHg)	77.00 (69.00 - 84.25)	80.50 (75.00 - 91.75)	-3.985	<0.001
FT3 (pmol/L)	4.96 (4.63 - 5.19)	4.94 (4.61 - 5.46)	-1.368	0.171
FT4 (pmol/L)	17.43 (15.96 - 19.19)	17.80 (16.50 - 19.90)	-0.681	0.496
TSH (uIU/mL)	2.28 (1.45 - 2.81)	1.54 (1.10 - 2.64)	-1.007	0.314
TPOAB (IU/mL)	14.44 (10.30 - 19.37)	12.55 (9.99 - 20.57)	-0.673	0.501
TGAB (IU/mL)	19.62 (15.81 - 56.31)	20.76 (15.42 - 48.62)	-0.256	0.798

SSL, serum selenium level; BMI, body mass index; SBP, systolic blood pressure; DBP, diastolic blood pressure yronine; FT4, free thyroxine; TSH, thyroid stimulating hormone; TPOAB, thyroidperoxidase antibodies; TGAB, thyroglobulin antibody.

Statistically significant variables in univariate analysis (serum selenium level, BMI, SBP and DBP) were included in multivariate analysis (**Table 7**). High serum selenium (*OR* = 0.320, 95%*CI*: 0.166 - 0.617) and DBP (*OR* = 1.066, 95%*CI*: 1.031 - 1.103) were related factors for the onset of multifocal tumors.

**Table 7:** Multivariate logistic retrospective analysis of factors related to unilateral and bilateral tumor incidence in PTC patients.

Subject	B	S.E.	Wald	*p*	*OR*	95%*CI*
SSL (μg/L)						
Low	-	-	-	-	1	-
Middle	-0.283	0.343	0.680	0.409	0.754	0.385 - 1.476
High	-1.139	0.335	11.586	0.001	0.320	0.166 - 0.617
BMI (kg/m^2^)	0.027	0.037	0.542	0.461	1.028	0.956 - 1.105
SBP (mmHg)	-0.011	0.011	0.905	0.341	0.989	0.967 - 1.012
DBP (mmHg)	0.064	0.017	13.689	<0.001	1.066	1.031 - 1.103

Serum selenium levels were categorized into three grades: Low: 51.3 - 74.5 μg/L (n = 90), Middle: 74.5 - 85.3 μg/L (n = 90), and High : 85.3 - 175.3 μg/L (n = 90). SSL, serum selenium level; BMI, body mass index; DBP, diastolic blood pressure.

## Discussion

In this study, the relationship between serum selenium levels and the pathological features of PTC was analyzed. The results showed: (1) Serum selenium levels of female PTC patients were lower than those of male PTC patients. (2) Serum selenium levels of PTC patients were negatively correlated with the number of lymph node metastasis, but there was no significant correlation between serum selenium levels and the stage and pathological subtype of PTC. (3) High serum selenium levels might be a protective factor in PTC patients.

Serum selenium levels were negatively correlated with the positive number of lymph node metastasis. First, the effects of selenium on cancer cells are highly concentration-dependent (22). Cancer cell growth was promoted by low to moderate serum selenium levels, while high serum selenium levels were cytotoxic and inhibited cancer cell growth (23). Second, as a crucial component of deiodinase, selenium plays a pivotal role in the conversion of T4 to T3 (24, 25). When there is a deficiency of selenium, it may lead to a reduction in the synthesis of thyroid hormones, potentially resulting in an elevation of thyroid stimulating hormone (TSH) levels (25). Thyroid cell growth can be promoted by TSH, and long-term high levels of TSH might induce and promote the occurrence and development of thyroid cancer. Therefore, low selenium might indirectly promote the increase of TSH and lead to the occurrence and development of tumors. However, the effect of selenium on TSH in humans has not been fully established. Rostami et al. observed a significant negative correlation (p < 0.001) between serum selenium levels and TSH in a low selenium population (26). Similarly, Tong et al. reported a significant negative correlation (p < 0.001) between serum selenium levels and TSH in healthy individuals (27). Our study did not find the association between serum selenium levels and TSH. Similarly, studies based on the NHANSE database in the United States have not found any significant association between serum selenium levels and TSH (28). Third, glutathione peroxidase 3 (GPX3), a selenoprotein, suppressed the metastasis of thyroid cancer by inhibiting the Wnt/β-catenin signaling pathway (29). Insufficient selenium levels might contribute to a reduction in GPX3 levels, thereby facilitating the metastasis of thyroid cancer. Notably, 81.69% of patients in our study demonstrated serum selenium levels below 90 ng/L. When the serum selenium concentration exceeds 90 ng/L, selenoproteins, including selenoprotein P and GPX, could be optimized; whereas, levels below this threshold are insufficient to achieve the optimal states of selenium proteins (30, 31).

High serum selenium levels might be a protective factor in PTC patients. At high selenium levels, serum selenium levels were negatively correlated to bilateral and multifocal tumors. First, hypermetabolism in tumor cells leads to increased reactive oxygen species (ROS), and these cells appear to be susceptible to oxidative stress (8, 9). The supplementation of selenium may exert an anti-tumor effect by inhibiting the ROS-mediated Akt/mTOR pathway (11). Furthermore, selenium induces cell death and apoptosis through superoxide generation in mitochondria and activation of the mitochondrial apoptotic pathway (32). Selenium could also affect tumor proliferation by affecting gene expression. Selenium inhibits tumor cell proliferation through DNA repair by p53-dependent effectors (13). In drug intervention experiments, it was shown that sodium selenite can significantly reduce cell viability and induce thyroid cancer cell G0/G1 cell cycle arrest and apoptosis in a dose-dependent manner (11). At the same time, the activities of T lymphocytes and NK cells were significantly enhanced after Se supplementation, and tumor proliferation could be suppressed (33). Notably, tumor cells have a higher metabolism than resting cells compared to healthy cells (34). In order to maintain the balance of the oxidation-antioxidation system, more antioxidant molecules such as selenoproteins need to be consumed. Therefore, the reduction of serum selenium levels might also be caused by the depletion process of tumorigenesis and development.

There was no significant association between serum selenium levels and PTC stage. This notion was also supported by a study of Korean women with thyroid cancer (35). However, the study by Jonklaas et al. showed that serum selenium levels might be negatively correlated with thyroid cancer stage (21). This discrepancy might be due to a variety of factors. First, the study population was limited to patients with PTC in our study, whereas in Jonklaas et al.’s study it was patients with differentiated thyroid cancer. Results might have been influenced by incorporation of other types of thyroid cancer. Second, despite serum selenium levels might be associated with thyroid cancer risk, randomized controlled trials on the effect of selenium supplementation on cancer have yielded inconsistent results (36). Serum selenium levels might be associated with some features of PTC, but not with tumor stage.

There was no significant difference in serum selenium levels among patients with different pathological subtypes of PTC. This is a preliminary exploration of serum selenium levels in patients with different subtypes of PTC. Similarly, no correlation was found between selenium and the tumor pathological subtype in the study of breast cancer (31). However, care needs to be taken that, due to the rarity of some subtypes, a sufficiently large sample size could not be obtained to improve the reliability of the evidence. Therefore, future studies with larger sample sizes need to be implemented.

The serum selenium levels of PTC patients were lower in females than males. Previous study found that there was no gender difference in serum selenium levels in Austrian (37). Similarly, the study in Western Romanesia revealed slightly lower selenium levels in young females compared to young males (38). However, some studies have found that females have lower serum selenium levels than males (39, 40). Notably, previous studies paid little attention to the differences in serum selenium levels between genders in PTC populations. The multi-pathway anti-tumor effects of selenium suggest that low selenium levels might serve as a susceptibility factor for PTC in female patients. In the future, studies on the relationship between diet and *in vivo* selenium metabolism need to be conducted in the PTC population, and gender differences should be taken into account.

This study has several limitations. First, as a cross-sectional study, a causal relationship between serum selenium levels and pathological features in PTC patients could not be established. Second, this study did not include a healthy control group, and there might be an insufficient incorporation of covariates. Third, patients positive for thyroid antibodies were not excluded during the inclusion process. Fourth, this study might have selective bias, because female PTC patients account for a relatively high proportion. Fifth, given the rarity of some subtypes of PTC, future studies with larger sample sizes are necessary to validate the reliability of the conclusions. Sixth, the relationship between selenium and pathological features in PTC patients has only been investigated at the serum level, thus further exploration of the protein molecular mechanism is warranted for a more comprehensive understanding.

## Conclusions

In conclusion, the serum selenium levels of PTC patients in females were lower than males. High serum selenium levels might be a protective factor in PTC patients. Therefore, further research is necessary in this aspect to better understand the influence of selenium on PTC progression.

## Data availability statement

The original contributions presented in the study are included in the article/**Supplementary Material**. Further inquiries can be directed to the corresponding authors.

## Ethics statement

The study was conducted in accordance with the Declaration of Helsinki, and approved by the Ethics Committee of the First Affiliated Hospital of Shandong First Medical University (Qianfoshan Hospital in Shandong Province) under the number YXLL-KY-2019031.

## Author contributions

SG: Document Retrieval, Data Extraction, Data analysis, Essay writing, and Paper submission. JZ, JY, HF and YT: Data analysis. YS, MS and JF: Data Extraction. JD and LL: Article innovation and Paper submission. All authors contributed to the article and approved the submitted version.
