# Supplementary material for: The association between serum selenium levels and pathological features of papillary thyroid cancer in 284 patients
Source: Front Endocrinol (Lausanne). 2023 Nov 3;14:1242250. doi: 10.3389/fendo.2023.1242250 (PMC10656612; doi:10.3389/fendo.2023.1242250)
Supplement: Supplementary file 1 [file DataSheet_1.docx]

| 1. 分组变量：性别 |
| --- |

| **Table S1 \|** Clinical data of PTC patients in different genders. | | | | | | | | |
| --- | --- | --- | --- | --- | --- | --- | --- | --- |
| Subject | | Male | | Female | | t/Z | *p* | |
| Number (%) | | 85 (29.93%) | | 199 (70.07%) | | - | - | |
| Age (years) | | 44.00 (34.50 - 52.50) | | 44.00 (34.50 - 52.50) | | -0.346 | 0.729 | |
| SSL (μg/L) | | 82.20 (74.80 - 88.65) | | 44.00 (34.50 - 52.50) | | -2.105 | 0.035 | |
| BMI (kg/m2) | | 27.71 ± 4.07 | | 25.38 ± 3.65 | | -4.702 | <0.001 | |
| Height (cm) | | 172.00 (170.00 - 177.00) | | 160.00 (158.00 - 165.00) | | -11.493 | <0.001 | |
| Weight (kg) | | 84.00 (73.50 - 92.00) | | 65.00 (59.00 - 72.50) | | -9.020 | <0.001 | |
| Waist (cm) | | 97.00 (92.00 - 103.50) | | 87.00 (81.00 - 95.50) | | -6.790 | <0.001 | |
| SBP (mmHg) | | 130.00 (120.00 - 140.00) | | 120.00 (110.00 - 132.00) | | -4.239 | <0.001 | |
| DBP (mmHg) | | 83.00 (77.00 - 94.00) | | 78.00 (72.00 - 85.00) | | -3.994 | <0.001 | |
| TPOAB (IU/mL) | | 11.99 (9.12 - 15.79) | | 14.53 (10.10 - 21.70) | | -2.538 | 0.011 | |
| TGAB (IU/mL) | | 17.76 (13.81 - 22.03) | | 23.27 (16.20 - 77.48) | | -4.739 | <0.001 | |
| SSL, serum selenium level; BMI, body mass index; SBP, systolic blood pressure; DBP, diastolic blood pressure; TPOAB, thyroidperoxidase antibodies; TGAB, thyroglobulin antibody. | | | | | | | | |
| **Table S2** **\|** Pearson correlation analysis of serum selenium levels and thyroid related indicators in PTC patients. | | | | | | |  |  |
| Subjects | | Serum selenium | | | | |  |  |
|  |  | r | | *p* | | |  |  |
| FT3 (pmol/L) | | 0.145 | | 0.254 | | |  |  |
| FT4 (pmol/L) | | 0.145 | | 0.253 | | |  |  |
| TSH (uIU/mL) | | 0.028 | | 0.825 | | |  |  |
| TPOAB (IU/mL) | | -0.049 | | 0.703 | | |  |  |
| TGAB (IU/mL) | | -0.005 | | 0.967 | | |  |  |
| FT3, free triiodothyronine; FT4, free thyroxine; TSH, thyroid stimulating hormone; TPOAB, thyroidperoxidase antibodies; TGAB, thyroglobulin antibody. | | | | | | |  |  |


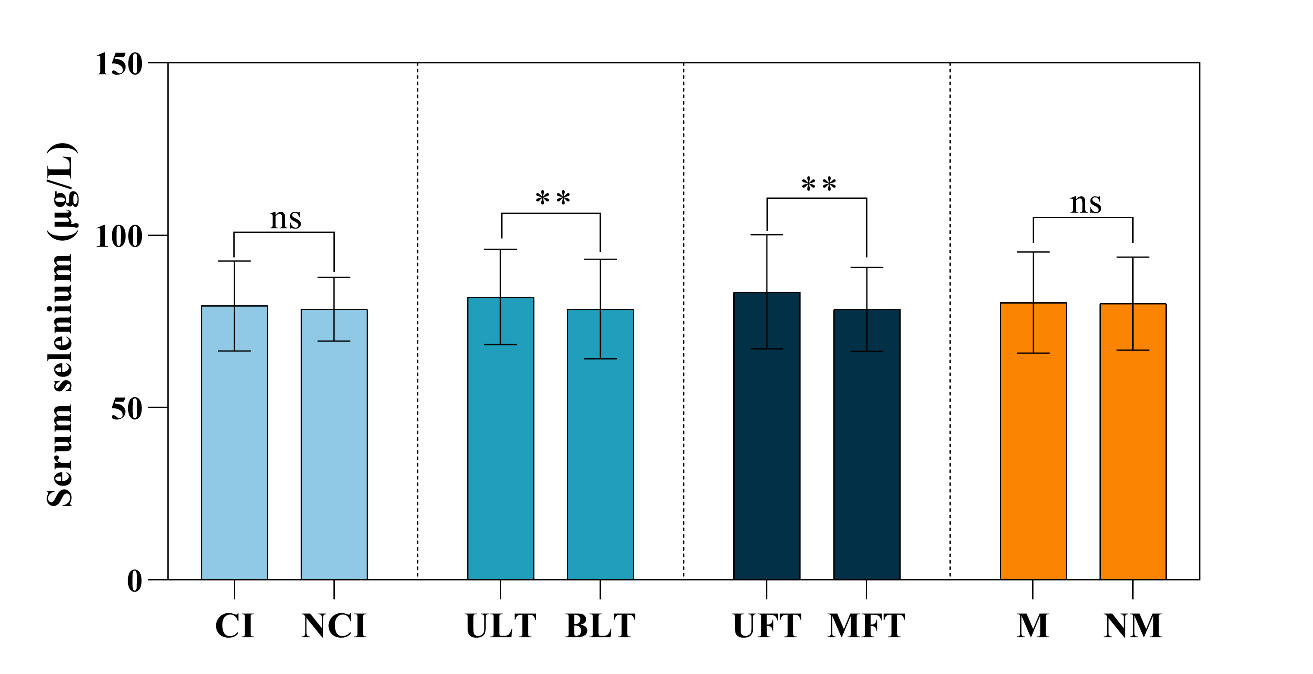


**Figure S1 |** Serum selenium levels among different pathological features in patients with CPTC (common papillary thyroid cancer). CI, capsule invasion; NCI, no capsule invasion; ULT, unilateral tumor; BLT, bilateral tumor; UFT, unifocal tumor; MFT, multifocal tumor; M, metastasis; NM, no metastasis.
